# 6-Meth­oxy­meth­oxy-7-trifluoro­methyl-1,3-benzodioxole-5-carbaldehyde

**DOI:** 10.1107/S1600536812022490

**Published:** 2012-06-02

**Authors:** Yanfeng Wang, Xinhui Pan, Dexiu Liu, Hong-Xiang Lou

**Affiliations:** a School of Pharmaceutical Sciences, Shandong University, Jinan 250012, People’s Republic of China; b Shuzhou Health College, Shuzhou 215009, People’s Republic of China

## Abstract

The title compound, C_11_H_9_F_3_O_5_, crystallizes with three mol­ecules in the asymmetric unit. One –CF_3_ group is disordered by rotation, with the F atoms split over two sets of sites with occupancies which converged to 0.888 (6) and 0.112 (6). Weak π–π inter­actions are observed between adjacent benzene rings [the shortest centroid–centroid distance is 3.8858 (4) Å], resulting in the formation of a supra­molecular chain along [100].

## Related literature

For the enhancement of the biological activity of F and CF_3_ containing mol­ecules compared to their unfluorinated analogues, see: Vrábel *et al.* (2007 ▶); Wilson & Danishefsky (2010 ▶); Bravo *et al.* (1994 ▶); Jung *et al.* (2002 ▶). For the synthesis of the title compound, an inter­mediate for the synthesis of biologic­ally active compounds, see: Corey *et al.* (1996 ▶); Weeratunga *et al.* (1987 ▶); Wu *et al.* (2004 ▶).
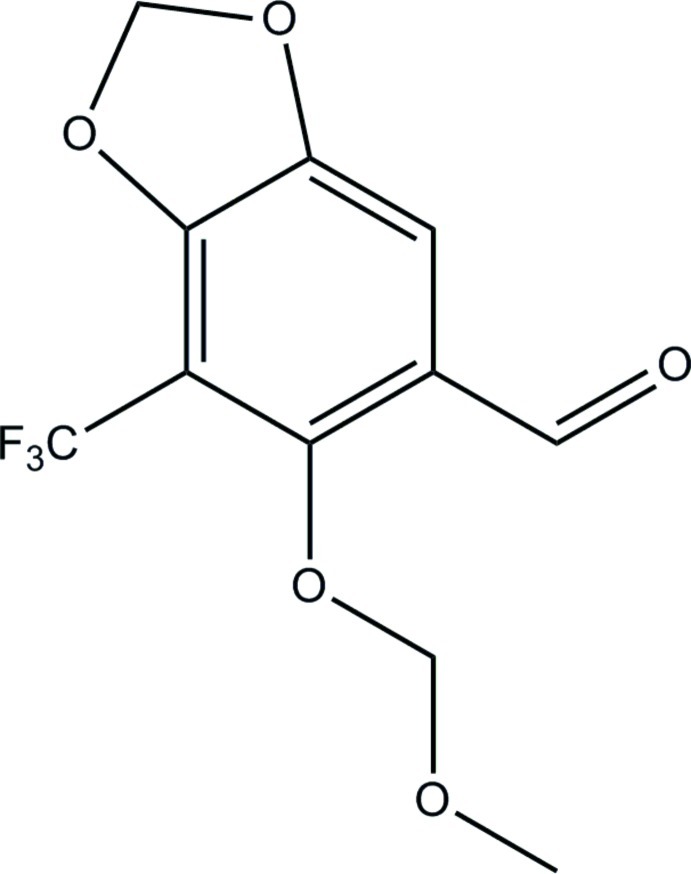


## Experimental

### 

#### Crystal data

C_11_H_9_F_3_O_5_
*M* *_r_* = 278.18
Triclinic, 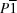

*a* = 9.3608 (10) Å
*b* = 12.6895 (14) Å
*c* = 14.6765 (16) Å
α = 97.007 (2)°
β = 93.625 (2)°
γ = 102.983 (2)°
*V* = 1678.7 (3) Å^3^
*Z* = 6
Mo *K*α radiation
μ = 0.16 mm^−1^
*T* = 173 K
0.10 × 0.10 × 0.10 mm

#### Data collection

Bruker APEXII CCD diffractometer
Absorption correction: multi-scan (*SADABS*; Bruker, 2005 ▶) *T*_min_ = 0.984, *T*_max_ = 0.984
8355 measured reflections
5842 independent reflections
4928 reflections with *I* > 2σ(*I*)
*R*_int_ = 0.021

#### Refinement

*R*[*F* ^2^ > 2σ(*F* ^2^)] = 0.041
*wR*(*F* ^2^) = 0.122
*S* = 1.03
5842 reflections
545 parameters
18 restraints
H-atom parameters constrained
Δρ_max_ = 0.28 e Å^−3^
Δρ_min_ = −0.28 e Å^−3^

### 

Data collection: *APEX2* (Bruker, 2005 ▶); cell refinement: *SAINT* (Bruker, 2005 ▶); data reduction: *SAINT*; program(s) used to solve structure: *SHELXS97* (Sheldrick, 2008 ▶); program(s) used to refine structure: *SHELXL97* (Sheldrick, 2008 ▶); molecular graphics: *DIAMOND* (Brandenburg, 2008 ▶); software used to prepare material for publication: *publCIF* (Westrip, 2010 ▶).

## Supplementary Material

Crystal structure: contains datablock(s) global, I. DOI: 10.1107/S1600536812022490/bh2420sup1.cif

Structure factors: contains datablock(s) I. DOI: 10.1107/S1600536812022490/bh2420Isup2.hkl

Supplementary material file. DOI: 10.1107/S1600536812022490/bh2420Isup3.cml

Additional supplementary materials:  crystallographic information; 3D view; checkCIF report

## Figures and Tables

**Fig. 1.**
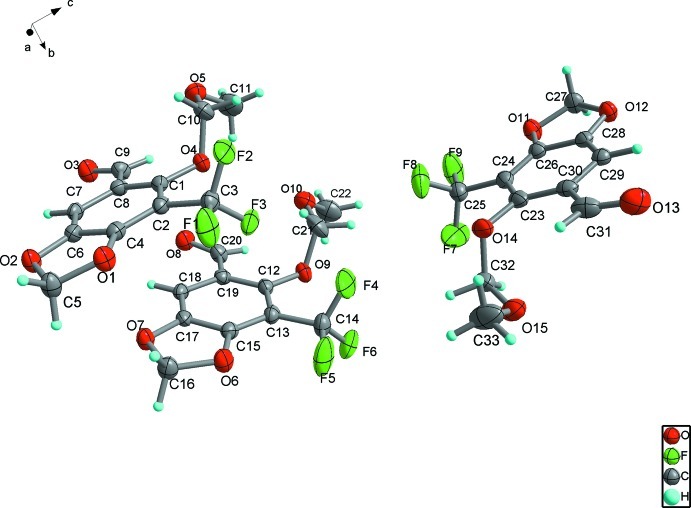
The asymmetric unit view of the title compound with displacement ellipsoids drawn at the 50% probability level. H atoms are represented as small spheres of arbitrary radii.

## supplementary crystallographic information

### Comment

In recent years, fluorinated compounds have been very important in the pharmaceutical field. Incorporation of a F atom instead of an H atom can alter the biological activity of a compound. Introduction of further F atoms in a CF_3_ group provides better lipophilicity, and the compounds might be pharmacologically more interesting compared to their non-fluorinated analogues (Vrábel *et al.*, 2007). Many heterocyclic compounds, which bear the trifluoromethyl group, are far superior to the parent compounds regarding their biological activity (Wilson & Danishefsky, 2010), including herbicides (Bravo *et al.*, 1994) and fungicides (Jung *et al.*, 2002). We report here the crystal structure of the title compound, which is used as an important starting material for the synthesis of a natural product derivative.

### Experimental

The title compound was synthesized according to literature methods (Corey *et al.*, 1996; Wu *et al.*, 2004; Weeratunga *et al.*, 1987). The resulting product was purified by column chromatography (silica gel, 200–300 meshes, eluent petroleum ether/ethyl acetate 10:1). The solid residue was crystallized by slow evaporation of an hexane/ethyl acetate (5:1) solution at room temperature. ^1^H-NMR (600 MHz, CDCl_3_) δ 3.62 (s, 3H, OCH_3_), 5.09 (s, 2H, CH_2_), 6.16 (s, 2H, ArOCH_2_), 7.42 (s, 1H, ArH), 10.14 (s, 1H, CHO); ^13^C-NMR (150 MHz, CDCl_3_) δ 58.11, 102, 103, 108, 123, 125, 145, 151, 155, 188; HRMS (EI^+^) m/*z*: calcd. for C_11_H_9_F_3_O_5_ (*M*^+^): 279.19, found: 279.19.

### Refinement

All H atoms were placed geometrically and treated as riding on their parent atoms with C—H = 0.98 (methyl), 0.95 (aromatic) or 0.99 Å (methylene), and *U*_iso_(H) = *x U*_eq_(C), with *x* = 1.5 for methyl groups and 1.2 for other H atoms. The CF_3_ group is disordered by rotation, and the F atoms are split over two sets of sites with occupancies of 0.888 (6) and 0.112 (6). Restraints were applied on these atoms.

### Crystal data

**Table:**

C_11_H_9_F_3_O_5_	*Z* = 6
*M**_r_* = 278.18	*F*(000) = 852
Triclinic, *P*1	*D*_x_ = 1.651 Mg m^−^^3^
Hall symbol: -P 1	Mo *K*α radiation, λ = 0.71073 Å
*a* = 9.3608 (10) Å	Cell parameters from 4637 reflections
*b* = 12.6895 (14) Å	θ = 2.8–27.5°
*c* = 14.6765 (16) Å	µ = 0.16 mm^−^^1^
α = 97.007 (2)°	*T* = 173 K
β = 93.625 (2)°	Block, colourless
γ = 102.983 (2)°	0.10 × 0.10 × 0.10 mm
*V* = 1678.7 (3) Å^3^	

### Data collection

**Table:**

Bruker APEXII CCD diffractometer	5842 independent reflections
Radiation source: fine-focus sealed tube	4928 reflections with *I* > 2σ(*I*)
Graphite monochromator	*R*_int_ = 0.021
φ and ω scans	θ_max_ = 25.0°, θ_min_ = 1.7°
Absorption correction: multi-scan (*SADABS*; Bruker, 2005)	*h* = −10→11
*T*_min_ = 0.984, *T*_max_ = 0.984	*k* = −15→10
8355 measured reflections	*l* = −17→16

### Refinement

**Table:**

Refinement on *F*^2^	Primary atom site location: structure-invariant direct methods
Least-squares matrix: full	Secondary atom site location: difference Fourier map
*R*[*F*^2^ > 2σ(*F*^2^)] = 0.041	Hydrogen site location: inferred from neighbouring sites
*wR*(*F*^2^) = 0.122	H-atom parameters constrained
*S* = 1.03	*w* = 1/[σ^2^(*F*_o_^2^) + (0.0634*P*)^2^ + 0.6455*P*] where *P* = (*F*_o_^2^ + 2*F*_c_^2^)/3
5842 reflections	(Δ/σ)_max_ = 0.002
545 parameters	Δρ_max_ = 0.28 e Å^−^^3^
18 restraints	Δρ_min_ = −0.28 e Å^−^^3^
0 constraints	

### Fractional atomic coordinates and isotropic or equivalent isotropic displacement parameters (Å^2^)

**Table:**

	*x*	*y*	*z*	*U*_iso_*/*U*_eq_	Occ. (<1)
C1	0.4148 (2)	0.44220 (14)	0.24627 (12)	0.0258 (4)	
C2	0.5685 (2)	0.46616 (14)	0.26506 (12)	0.0259 (4)	
C3	0.6377 (2)	0.50456 (16)	0.36188 (13)	0.0322 (4)	
C4	0.6498 (2)	0.45487 (14)	0.19079 (12)	0.0255 (4)	
C5	0.8251 (2)	0.46037 (18)	0.09341 (13)	0.0340 (4)	
H5A	0.8685	0.5324	0.0751	0.041*	
H5B	0.8944	0.4126	0.0825	0.041*	
C6	0.5815 (2)	0.41995 (14)	0.10140 (12)	0.0253 (4)	
C7	0.4337 (2)	0.39694 (14)	0.08251 (12)	0.0254 (4)	
H7	0.3894	0.3732	0.0212	0.030*	
C8	0.3471 (2)	0.40933 (14)	0.15670 (12)	0.0261 (4)	
C9	0.1878 (2)	0.39213 (16)	0.13658 (13)	0.0325 (4)	
H9	0.1311	0.4092	0.1854	0.039*	
C10	0.2684 (2)	0.36310 (17)	0.36009 (14)	0.0368 (5)	
H10A	0.3165	0.3032	0.3405	0.044*	
H10B	0.2849	0.3801	0.4281	0.044*	
C11	0.0396 (3)	0.4048 (2)	0.3714 (2)	0.0561 (7)	
H11A	−0.0659	0.3744	0.3546	0.084*	
H11B	0.0701	0.4737	0.3463	0.084*	
H11C	0.0591	0.4183	0.4387	0.084*	
C12	0.4460 (2)	0.79048 (15)	0.33160 (12)	0.0260 (4)	
C13	0.6003 (2)	0.82136 (15)	0.34819 (12)	0.0278 (4)	
C14	0.6724 (2)	0.88593 (18)	0.43803 (14)	0.0377 (5)	
C15	0.6789 (2)	0.78481 (15)	0.27987 (12)	0.0261 (4)	
C16	0.8521 (2)	0.74583 (17)	0.19183 (14)	0.0350 (5)	
H16A	0.9085	0.7991	0.1557	0.042*	
H16B	0.9093	0.6907	0.2018	0.042*	
C17	0.6090 (2)	0.72025 (14)	0.19871 (12)	0.0242 (4)	
C18	0.4606 (2)	0.69090 (14)	0.18152 (12)	0.0257 (4)	
H18	0.4149	0.6471	0.1256	0.031*	
C19	0.3766 (2)	0.72763 (14)	0.24954 (12)	0.0253 (4)	
C20	0.2158 (2)	0.70242 (17)	0.23068 (13)	0.0327 (4)	
H20	0.1615	0.7387	0.2713	0.039*	
C21	0.3062 (3)	0.7483 (2)	0.45855 (15)	0.0446 (6)	
H21A	0.3602	0.6894	0.4546	0.054*	
H21B	0.3214	0.7851	0.5230	0.054*	
C22	0.0704 (3)	0.7777 (2)	0.4588 (2)	0.0585 (7)	
H22A	−0.0336	0.7405	0.4440	0.088*	
H22B	0.0947	0.8390	0.4234	0.088*	
H22C	0.0887	0.8052	0.5248	0.088*	
C23	0.5757 (2)	0.86851 (15)	0.85712 (13)	0.0308 (4)	
C24	0.4215 (2)	0.85065 (15)	0.84339 (13)	0.0306 (4)	
C25	0.3445 (3)	0.84013 (18)	0.74898 (14)	0.0396 (5)	
C26	0.3451 (2)	0.84472 (14)	0.92068 (13)	0.0281 (4)	
C27	0.1749 (2)	0.82327 (18)	1.02165 (13)	0.0353 (5)	
H27A	0.1154	0.8752	1.0431	0.042*	
H27B	0.1220	0.7488	1.0302	0.042*	
C28	0.4178 (2)	0.85820 (14)	1.00856 (12)	0.0263 (4)	
C29	0.5655 (2)	0.87629 (14)	1.02279 (13)	0.0282 (4)	
H29	0.6132	0.8862	1.0834	0.034*	
C30	0.6474 (2)	0.88013 (15)	0.94515 (13)	0.0300 (4)	
C31	0.8066 (2)	0.89239 (17)	0.96167 (17)	0.0410 (5)	
H31	0.8616	0.8853	0.9099	0.049*	
C32	0.7103 (3)	0.97212 (17)	0.75019 (14)	0.0423 (5)	
H32A	0.7049	0.9627	0.6820	0.051*	
H32B	0.6487	1.0232	0.7702	0.051*	
C33	0.9564 (3)	0.9601 (2)	0.7452 (2)	0.0651 (8)	
H33A	1.0551	0.9917	0.7766	0.098*	
H33B	0.9573	0.9680	0.6796	0.098*	
H33C	0.9271	0.8825	0.7519	0.098*	
F1	0.78370 (14)	0.52599 (14)	0.36687 (9)	0.0609 (4)	
F2	0.59564 (16)	0.43130 (10)	0.41881 (8)	0.0491 (3)	
F3	0.60334 (15)	0.59570 (9)	0.39913 (8)	0.0439 (3)	
F4	0.63938 (19)	0.83087 (13)	0.50907 (9)	0.0677 (5)	
F5	0.81807 (15)	0.91193 (15)	0.44024 (11)	0.0737 (5)	
F6	0.63210 (16)	0.97844 (11)	0.45818 (9)	0.0548 (4)	
F7	0.3831 (3)	0.93019 (15)	0.71022 (16)	0.0706 (9)	0.888 (6)
F7B	0.302 (3)	0.9299 (16)	0.7436 (13)	0.074 (6)	0.112 (6)
F8	0.3705 (3)	0.7592 (2)	0.69059 (10)	0.0583 (7)	0.888 (6)
F8B	0.431 (2)	0.827 (2)	0.6842 (10)	0.067 (5)	0.112 (6)
F9	0.1981 (2)	0.8201 (3)	0.74828 (13)	0.0635 (7)	0.888 (6)
F9B	0.231 (2)	0.7631 (17)	0.7437 (11)	0.059 (5)	0.112 (6)
O1	0.79772 (14)	0.47249 (11)	0.18950 (9)	0.0330 (3)	
O2	0.68690 (14)	0.41280 (11)	0.04150 (9)	0.0324 (3)	
O3	0.12449 (15)	0.35701 (13)	0.06032 (10)	0.0409 (4)	
O4	0.33447 (15)	0.45868 (11)	0.31963 (9)	0.0315 (3)	
O5	0.12040 (16)	0.32932 (12)	0.33439 (10)	0.0430 (4)	
O6	0.82681 (14)	0.80063 (12)	0.27905 (9)	0.0368 (3)	
O7	0.71247 (14)	0.69427 (11)	0.14359 (9)	0.0320 (3)	
O8	0.14850 (15)	0.63790 (12)	0.16616 (10)	0.0402 (4)	
O9	0.36529 (15)	0.82610 (11)	0.39864 (9)	0.0332 (3)	
O10	0.15914 (18)	0.70333 (13)	0.43583 (11)	0.0484 (4)	
O11	0.19842 (15)	0.82781 (12)	0.92587 (9)	0.0376 (3)	
O12	0.31665 (14)	0.85179 (11)	1.07222 (9)	0.0321 (3)	
O13	0.87177 (16)	0.91108 (14)	1.03801 (12)	0.0492 (4)	
O14	0.65266 (17)	0.86765 (11)	0.78023 (10)	0.0403 (4)	
O15	0.85359 (18)	1.01619 (12)	0.78551 (11)	0.0475 (4)	

### Atomic displacement parameters (Å^2^)

**Table:**

	*U*^11^	*U*^22^	*U*^33^	*U*^12^	*U*^13^	*U*^23^
C1	0.0318 (10)	0.0228 (9)	0.0231 (9)	0.0070 (7)	0.0041 (7)	0.0030 (7)
C2	0.0323 (10)	0.0223 (9)	0.0223 (9)	0.0061 (7)	−0.0012 (7)	0.0028 (7)
C3	0.0376 (11)	0.0331 (10)	0.0251 (10)	0.0095 (9)	−0.0019 (8)	0.0012 (8)
C4	0.0261 (9)	0.0228 (9)	0.0270 (9)	0.0049 (7)	−0.0013 (7)	0.0040 (7)
C5	0.0247 (10)	0.0436 (12)	0.0313 (10)	0.0075 (9)	0.0026 (8)	−0.0035 (9)
C6	0.0287 (10)	0.0223 (9)	0.0237 (9)	0.0041 (7)	0.0028 (7)	0.0018 (7)
C7	0.0296 (10)	0.0237 (9)	0.0208 (9)	0.0040 (7)	−0.0016 (7)	0.0010 (7)
C8	0.0269 (10)	0.0233 (9)	0.0266 (9)	0.0032 (7)	0.0006 (7)	0.0037 (7)
C9	0.0303 (10)	0.0374 (11)	0.0290 (10)	0.0057 (8)	0.0030 (8)	0.0060 (8)
C10	0.0416 (12)	0.0398 (11)	0.0304 (10)	0.0076 (9)	0.0090 (9)	0.0113 (9)
C11	0.0421 (14)	0.0574 (15)	0.0715 (17)	0.0114 (12)	0.0225 (12)	0.0110 (13)
C12	0.0320 (10)	0.0260 (9)	0.0216 (9)	0.0095 (8)	0.0053 (7)	0.0037 (7)
C13	0.0333 (10)	0.0260 (9)	0.0229 (9)	0.0066 (8)	−0.0004 (8)	0.0012 (7)
C14	0.0393 (12)	0.0420 (12)	0.0280 (10)	0.0080 (9)	−0.0018 (9)	−0.0049 (9)
C15	0.0258 (9)	0.0259 (9)	0.0257 (9)	0.0040 (7)	0.0004 (7)	0.0049 (7)
C16	0.0259 (10)	0.0394 (11)	0.0363 (11)	0.0050 (8)	0.0046 (8)	−0.0034 (9)
C17	0.0291 (10)	0.0234 (9)	0.0203 (9)	0.0055 (7)	0.0047 (7)	0.0036 (7)
C18	0.0293 (10)	0.0244 (9)	0.0219 (9)	0.0039 (7)	−0.0008 (7)	0.0029 (7)
C19	0.0264 (9)	0.0266 (9)	0.0234 (9)	0.0058 (7)	0.0024 (7)	0.0061 (7)
C20	0.0291 (10)	0.0427 (11)	0.0270 (10)	0.0087 (9)	0.0045 (8)	0.0059 (9)
C21	0.0489 (14)	0.0646 (15)	0.0299 (11)	0.0241 (12)	0.0136 (10)	0.0188 (10)
C22	0.0427 (14)	0.0707 (18)	0.0671 (17)	0.0150 (13)	0.0168 (12)	0.0199 (14)
C23	0.0410 (11)	0.0205 (9)	0.0317 (10)	0.0068 (8)	0.0114 (8)	0.0032 (8)
C24	0.0410 (11)	0.0240 (9)	0.0263 (10)	0.0075 (8)	0.0019 (8)	0.0023 (7)
C25	0.0499 (14)	0.0379 (12)	0.0304 (11)	0.0103 (10)	0.0011 (10)	0.0043 (9)
C26	0.0305 (10)	0.0224 (9)	0.0310 (10)	0.0064 (7)	0.0004 (8)	0.0033 (7)
C27	0.0286 (10)	0.0443 (12)	0.0326 (10)	0.0080 (9)	0.0039 (8)	0.0044 (9)
C28	0.0310 (10)	0.0208 (9)	0.0268 (9)	0.0049 (7)	0.0036 (8)	0.0037 (7)
C29	0.0322 (10)	0.0222 (9)	0.0293 (10)	0.0044 (8)	−0.0004 (8)	0.0058 (7)
C30	0.0325 (10)	0.0214 (9)	0.0362 (11)	0.0048 (8)	0.0055 (8)	0.0060 (8)
C31	0.0351 (11)	0.0374 (12)	0.0532 (14)	0.0076 (9)	0.0125 (10)	0.0149 (10)
C32	0.0579 (14)	0.0348 (11)	0.0305 (11)	0.0005 (10)	0.0124 (10)	0.0057 (9)
C33	0.0581 (16)	0.0467 (14)	0.086 (2)	0.0018 (12)	0.0384 (15)	−0.0041 (14)
F1	0.0348 (7)	0.1043 (12)	0.0347 (7)	0.0138 (7)	−0.0097 (6)	−0.0128 (7)
F2	0.0797 (10)	0.0418 (7)	0.0255 (6)	0.0151 (7)	−0.0074 (6)	0.0086 (5)
F3	0.0665 (9)	0.0327 (6)	0.0298 (6)	0.0150 (6)	−0.0069 (6)	−0.0059 (5)
F4	0.1021 (12)	0.0654 (10)	0.0266 (7)	0.0079 (9)	−0.0151 (7)	0.0041 (6)
F5	0.0385 (8)	0.1045 (13)	0.0585 (9)	0.0084 (8)	−0.0117 (7)	−0.0416 (9)
F6	0.0660 (9)	0.0422 (7)	0.0490 (8)	0.0134 (7)	−0.0026 (7)	−0.0187 (6)
F7	0.100 (2)	0.0513 (11)	0.0553 (13)	0.0039 (11)	−0.0237 (12)	0.0275 (9)
F7B	0.090 (10)	0.087 (9)	0.060 (8)	0.041 (8)	0.001 (7)	0.027 (7)
F8	0.0898 (16)	0.0586 (14)	0.0277 (8)	0.0307 (12)	−0.0052 (8)	−0.0091 (8)
F8B	0.071 (8)	0.080 (10)	0.044 (7)	0.015 (7)	−0.002 (6)	−0.001 (6)
F9	0.0491 (11)	0.102 (2)	0.0359 (9)	0.0194 (12)	−0.0100 (7)	−0.0001 (11)
F9B	0.042 (7)	0.065 (9)	0.056 (7)	−0.011 (6)	−0.016 (6)	0.012 (7)
O1	0.0248 (7)	0.0443 (8)	0.0283 (7)	0.0074 (6)	−0.0019 (5)	0.0019 (6)
O2	0.0266 (7)	0.0425 (8)	0.0255 (7)	0.0059 (6)	0.0037 (5)	−0.0018 (6)
O3	0.0273 (7)	0.0540 (9)	0.0351 (8)	0.0020 (7)	−0.0033 (6)	−0.0017 (7)
O4	0.0358 (7)	0.0327 (7)	0.0260 (7)	0.0067 (6)	0.0094 (6)	0.0026 (5)
O5	0.0402 (9)	0.0381 (8)	0.0459 (9)	0.0005 (7)	0.0076 (7)	0.0018 (7)
O6	0.0254 (7)	0.0481 (9)	0.0310 (7)	0.0023 (6)	0.0004 (6)	−0.0042 (6)
O7	0.0250 (7)	0.0414 (8)	0.0275 (7)	0.0067 (6)	0.0041 (5)	−0.0026 (6)
O8	0.0291 (7)	0.0477 (9)	0.0380 (8)	0.0017 (6)	−0.0017 (6)	−0.0002 (7)
O9	0.0367 (8)	0.0392 (8)	0.0259 (7)	0.0136 (6)	0.0089 (6)	0.0018 (6)
O10	0.0526 (10)	0.0443 (9)	0.0478 (9)	0.0061 (7)	0.0148 (8)	0.0091 (7)
O11	0.0298 (7)	0.0524 (9)	0.0307 (7)	0.0103 (6)	−0.0004 (6)	0.0070 (6)
O12	0.0287 (7)	0.0409 (8)	0.0264 (7)	0.0072 (6)	0.0041 (5)	0.0044 (6)
O13	0.0317 (8)	0.0565 (10)	0.0582 (11)	0.0059 (7)	−0.0002 (7)	0.0138 (8)
O14	0.0552 (9)	0.0282 (7)	0.0364 (8)	0.0045 (6)	0.0207 (7)	0.0017 (6)
O15	0.0506 (10)	0.0347 (8)	0.0508 (9)	−0.0011 (7)	0.0165 (8)	−0.0044 (7)

### Geometric parameters (Å, º)

**Table:**

C1—O4	1.373 (2)	C18—C19	1.408 (3)
C1—C8	1.395 (3)	C18—H18	0.9500
C1—C2	1.404 (3)	C19—C20	1.467 (3)
C2—C4	1.380 (3)	C20—O8	1.212 (2)
C2—C3	1.500 (2)	C20—H20	0.9500
C3—F1	1.327 (2)	C21—O10	1.369 (3)
C3—F3	1.332 (2)	C21—O9	1.443 (2)
C3—F2	1.336 (2)	C21—H21A	0.9900
C4—O1	1.354 (2)	C21—H21B	0.9900
C4—C6	1.397 (3)	C22—O10	1.416 (3)
C5—O2	1.425 (2)	C22—H22A	0.9800
C5—O1	1.445 (2)	C22—H22B	0.9800
C5—H5A	0.9900	C22—H22C	0.9800
C5—H5B	0.9900	C23—O14	1.377 (2)
C6—C7	1.352 (3)	C23—C30	1.393 (3)
C6—O2	1.373 (2)	C23—C24	1.407 (3)
C7—C8	1.412 (3)	C24—C26	1.379 (3)
C7—H7	0.9500	C24—C25	1.497 (3)
C8—C9	1.464 (3)	C25—F9B	1.266 (15)
C9—O3	1.216 (2)	C25—F8B	1.304 (16)
C9—H9	0.9500	C25—F7B	1.300 (18)
C10—O5	1.368 (2)	C25—F7	1.326 (3)
C10—O4	1.446 (2)	C25—F9	1.336 (3)
C10—H10A	0.9900	C25—F8	1.331 (3)
C10—H10B	0.9900	C26—O11	1.350 (2)
C11—O5	1.427 (3)	C26—C28	1.392 (3)
C11—H11A	0.9800	C27—O12	1.427 (2)
C11—H11B	0.9800	C27—O11	1.443 (2)
C11—H11C	0.9800	C27—H27A	0.9900
C12—O9	1.372 (2)	C27—H27B	0.9900
C12—C19	1.394 (3)	C28—C29	1.348 (3)
C12—C13	1.406 (3)	C28—O12	1.368 (2)
C13—C15	1.377 (3)	C29—C30	1.413 (3)
C13—C14	1.497 (3)	C29—H29	0.9500
C14—F6	1.318 (3)	C30—C31	1.465 (3)
C14—F5	1.326 (3)	C31—O13	1.211 (3)
C14—F4	1.339 (3)	C31—H31	0.9500
C15—O6	1.355 (2)	C32—O15	1.373 (3)
C15—C17	1.393 (3)	C32—O14	1.447 (2)
C16—O7	1.422 (2)	C32—H32A	0.9900
C16—O6	1.440 (2)	C32—H32B	0.9900
C16—H16A	0.9900	C33—O15	1.435 (3)
C16—H16B	0.9900	C33—H33A	0.9800
C17—C18	1.353 (3)	C33—H33B	0.9800
C17—O7	1.371 (2)	C33—H33C	0.9800
			
O4—C1—C8	121.40 (16)	O8—C20—C19	123.64 (18)
O4—C1—C2	116.80 (15)	O8—C20—H20	118.2
C8—C1—C2	121.70 (16)	C19—C20—H20	118.2
C4—C2—C1	116.89 (16)	O10—C21—O9	112.04 (17)
C4—C2—C3	122.85 (17)	O10—C21—H21A	109.2
C1—C2—C3	120.25 (16)	O9—C21—H21A	109.2
F1—C3—F3	106.24 (16)	O10—C21—H21B	109.2
F1—C3—F2	106.72 (16)	O9—C21—H21B	109.2
F3—C3—F2	105.70 (16)	H21A—C21—H21B	107.9
F1—C3—C2	112.31 (16)	O10—C22—H22A	109.5
F3—C3—C2	112.74 (16)	O10—C22—H22B	109.5
F2—C3—C2	112.61 (16)	H22A—C22—H22B	109.5
O1—C4—C2	128.95 (16)	O10—C22—H22C	109.5
O1—C4—C6	109.80 (16)	H22A—C22—H22C	109.5
C2—C4—C6	121.24 (17)	H22B—C22—H22C	109.5
O2—C5—O1	107.09 (14)	O14—C23—C30	120.96 (18)
O2—C5—H5A	110.3	O14—C23—C24	117.75 (17)
O1—C5—H5A	110.3	C30—C23—C24	121.18 (17)
O2—C5—H5B	110.3	C26—C24—C23	117.07 (17)
O1—C5—H5B	110.3	C26—C24—C25	121.70 (18)
H5A—C5—H5B	108.6	C23—C24—C25	121.22 (18)
C7—C6—O2	128.38 (16)	F9B—C25—F8B	113.5 (12)
C7—C6—C4	122.35 (17)	F9B—C25—F7B	107.7 (12)
O2—C6—C4	109.26 (16)	F8B—C25—F7B	109.0 (12)
C6—C7—C8	117.95 (16)	F7—C25—F9	105.5 (2)
C6—C7—H7	121.0	F7—C25—F8	106.5 (2)
C8—C7—H7	121.0	F9—C25—F8	105.2 (2)
C1—C8—C7	119.84 (17)	F7—C25—C24	112.52 (18)
C1—C8—C9	121.52 (17)	F9—C25—C24	113.25 (18)
C7—C8—C9	118.57 (16)	F8—C25—C24	113.20 (18)
O3—C9—C8	123.04 (18)	O11—C26—C24	128.49 (17)
O3—C9—H9	118.5	O11—C26—C28	110.13 (16)
C8—C9—H9	118.5	C24—C26—C28	121.38 (18)
O5—C10—O4	111.60 (16)	O12—C27—O11	106.96 (15)
O5—C10—H10A	109.3	O12—C27—H27A	110.3
O4—C10—H10A	109.3	O11—C27—H27A	110.3
O5—C10—H10B	109.3	O12—C27—H27B	110.3
O4—C10—H10B	109.3	O11—C27—H27B	110.3
H10A—C10—H10B	108.0	H27A—C27—H27B	108.6
O5—C11—H11A	109.5	C29—C28—O12	128.65 (17)
O5—C11—H11B	109.5	C29—C28—C26	122.17 (17)
H11A—C11—H11B	109.5	O12—C28—C26	109.17 (16)
O5—C11—H11C	109.5	C28—C29—C30	118.16 (17)
H11A—C11—H11C	109.5	C28—C29—H29	120.9
H11B—C11—H11C	109.5	C30—C29—H29	120.9
O9—C12—C19	120.80 (16)	C23—C30—C29	120.00 (18)
O9—C12—C13	117.59 (16)	C23—C30—C31	122.72 (18)
C19—C12—C13	121.61 (16)	C29—C30—C31	117.23 (18)
C15—C13—C12	116.53 (16)	O13—C31—C30	123.3 (2)
C15—C13—C14	122.72 (17)	O13—C31—H31	118.4
C12—C13—C14	120.69 (17)	C30—C31—H31	118.4
F6—C14—F5	106.30 (18)	O15—C32—O14	111.72 (18)
F6—C14—F4	105.33 (17)	O15—C32—H32A	109.3
F5—C14—F4	106.69 (18)	O14—C32—H32A	109.3
F6—C14—C13	113.57 (17)	O15—C32—H32B	109.3
F5—C14—C13	112.56 (17)	O14—C32—H32B	109.3
F4—C14—C13	111.84 (17)	H32A—C32—H32B	107.9
O6—C15—C13	128.50 (16)	O15—C33—H33A	109.5
O6—C15—C17	109.80 (16)	O15—C33—H33B	109.5
C13—C15—C17	121.68 (17)	H33A—C33—H33B	109.5
O7—C16—O6	107.78 (15)	O15—C33—H33C	109.5
O7—C16—H16A	110.2	H33A—C33—H33C	109.5
O6—C16—H16A	110.2	H33B—C33—H33C	109.5
O7—C16—H16B	110.2	C4—O1—C5	106.13 (13)
O6—C16—H16B	110.2	C6—O2—C5	106.07 (14)
H16A—C16—H16B	108.5	C1—O4—C10	116.60 (14)
C18—C17—O7	128.01 (16)	C10—O5—C11	112.88 (17)
C18—C17—C15	122.36 (17)	C15—O6—C16	106.43 (14)
O7—C17—C15	109.63 (15)	C17—O7—C16	106.33 (14)
C17—C18—C19	117.56 (16)	C12—O9—C21	116.13 (15)
C17—C18—H18	121.2	C21—O10—C22	112.81 (19)
C19—C18—H18	121.2	C26—O11—C27	106.53 (14)
C12—C19—C18	120.25 (16)	C28—O12—C27	106.64 (14)
C12—C19—C20	120.90 (17)	C23—O14—C32	116.82 (15)
C18—C19—C20	118.80 (16)	C32—O15—C33	113.45 (18)
			
O4—C1—C2—C4	−176.86 (15)	C26—C24—C25—F9B	−42.5 (13)
C8—C1—C2—C4	−0.5 (3)	C23—C24—C25—F9B	138.4 (12)
O4—C1—C2—C3	1.8 (2)	C26—C24—C25—F8B	−167.6 (12)
C8—C1—C2—C3	178.13 (16)	C23—C24—C25—F8B	13.2 (12)
C4—C2—C3—F1	0.1 (3)	C26—C24—C25—F7B	72.8 (13)
C1—C2—C3—F1	−178.42 (17)	C23—C24—C25—F7B	−106.4 (13)
C4—C2—C3—F3	120.10 (19)	C26—C24—C25—F7	116.7 (3)
C1—C2—C3—F3	−58.4 (2)	C23—C24—C25—F7	−62.4 (3)
C4—C2—C3—F2	−120.39 (19)	C26—C24—C25—F9	−2.8 (3)
C1—C2—C3—F2	61.1 (2)	C23—C24—C25—F9	178.0 (2)
C1—C2—C4—O1	179.94 (17)	C26—C24—C25—F8	−122.5 (2)
C3—C2—C4—O1	1.4 (3)	C23—C24—C25—F8	58.3 (3)
C1—C2—C4—C6	−0.7 (3)	C23—C24—C26—O11	−179.62 (17)
C3—C2—C4—C6	−179.32 (17)	C25—C24—C26—O11	1.2 (3)
O1—C4—C6—C7	−179.61 (16)	C23—C24—C26—C28	1.4 (3)
C2—C4—C6—C7	0.9 (3)	C25—C24—C26—C28	−177.84 (17)
O1—C4—C6—O2	1.2 (2)	O11—C26—C28—C29	179.98 (16)
C2—C4—C6—O2	−178.25 (16)	C24—C26—C28—C29	−0.8 (3)
O2—C6—C7—C8	179.14 (17)	O11—C26—C28—O12	−1.0 (2)
C4—C6—C7—C8	0.1 (3)	C24—C26—C28—O12	178.14 (16)
O4—C1—C8—C7	177.74 (16)	O12—C28—C29—C30	−179.55 (17)
C2—C1—C8—C7	1.5 (3)	C26—C28—C29—C30	−0.8 (3)
O4—C1—C8—C9	0.7 (3)	O14—C23—C30—C29	−177.40 (16)
C2—C1—C8—C9	−175.48 (17)	C24—C23—C30—C29	−1.3 (3)
C6—C7—C8—C1	−1.3 (3)	O14—C23—C30—C31	0.2 (3)
C6—C7—C8—C9	175.79 (16)	C24—C23—C30—C31	176.32 (17)
C1—C8—C9—O3	−175.27 (18)	C28—C29—C30—C23	1.8 (3)
C7—C8—C9—O3	7.7 (3)	C28—C29—C30—C31	−175.92 (17)
O9—C12—C13—C15	−179.89 (15)	C23—C30—C31—O13	174.0 (2)
C19—C12—C13—C15	−1.0 (3)	C29—C30—C31—O13	−8.3 (3)
O9—C12—C13—C14	3.1 (3)	C2—C4—O1—C5	−173.78 (18)
C19—C12—C13—C14	−178.07 (17)	C6—C4—O1—C5	6.8 (2)
C15—C13—C14—F6	126.8 (2)	O2—C5—O1—C4	−12.1 (2)
C12—C13—C14—F6	−56.4 (3)	C7—C6—O2—C5	172.08 (18)
C15—C13—C14—F5	5.9 (3)	C4—C6—O2—C5	−8.79 (19)
C12—C13—C14—F5	−177.21 (18)	O1—C5—O2—C6	12.8 (2)
C15—C13—C14—F4	−114.2 (2)	C8—C1—O4—C10	83.8 (2)
C12—C13—C14—F4	62.7 (2)	C2—C1—O4—C10	−99.78 (19)
C12—C13—C15—O6	−178.52 (17)	O5—C10—O4—C1	−103.73 (19)
C14—C13—C15—O6	−1.5 (3)	O4—C10—O5—C11	−67.4 (2)
C12—C13—C15—C17	−0.2 (3)	C13—C15—O6—C16	−179.92 (19)
C14—C13—C15—C17	176.76 (18)	C17—C15—O6—C16	1.6 (2)
O6—C15—C17—C18	179.50 (16)	O7—C16—O6—C15	−2.1 (2)
C13—C15—C17—C18	0.9 (3)	C18—C17—O7—C16	179.16 (18)
O6—C15—C17—O7	−0.6 (2)	C15—C17—O7—C16	−0.75 (19)
C13—C15—C17—O7	−179.15 (16)	O6—C16—O7—C17	1.7 (2)
O7—C17—C18—C19	179.76 (16)	C19—C12—O9—C21	81.4 (2)
C15—C17—C18—C19	−0.3 (3)	C13—C12—O9—C21	−99.8 (2)
O9—C12—C19—C18	−179.54 (15)	O10—C21—O9—C12	−102.7 (2)
C13—C12—C19—C18	1.6 (3)	O9—C21—O10—C22	−71.9 (2)
O9—C12—C19—C20	3.1 (3)	C24—C26—O11—C27	177.13 (19)
C13—C12—C19—C20	−175.78 (17)	C28—C26—O11—C27	−3.8 (2)
C17—C18—C19—C12	−0.9 (3)	O12—C27—O11—C26	7.0 (2)
C17—C18—C19—C20	176.55 (16)	C29—C28—O12—C27	−175.68 (19)
C12—C19—C20—O8	−170.82 (18)	C26—C28—O12—C27	5.4 (2)
C18—C19—C20—O8	11.7 (3)	O11—C27—O12—C28	−7.6 (2)
O14—C23—C24—C26	175.93 (16)	C30—C23—O14—C32	−89.6 (2)
C30—C23—C24—C26	−0.3 (3)	C24—C23—O14—C32	94.2 (2)
O14—C23—C24—C25	−4.9 (3)	O15—C32—O14—C23	94.5 (2)
C30—C23—C24—C25	178.90 (17)	O14—C32—O15—C33	71.4 (2)
